# Optimization of Electrospray Ionization by Statistical Design of Experiments and Response Surface Methodology: Protein–Ligand Equilibrium Dissociation Constant Determinations

**DOI:** 10.1007/s13361-016-1417-x

**Published:** 2016-05-25

**Authors:** Liliana Pedro, Wesley C. Van Voorhis, Ronald J. Quinn

**Affiliations:** 1 Eskitis Institute for Drug Discovery, Griffith University, Brisbane, Queensland Australia; 2 Department of Medicine, University of Washington, Seattle, WA USA

**Keywords:** Kd, ESI-FTMS, Plasmodium vivax Guanylate Kinase (PvGK), Protein-ligand complex

## Abstract

Electrospray ionization mass spectrometry (ESI-MS) binding studies between proteins and ligands under native conditions require that instrumental ESI source conditions are optimized if relative solution-phase equilibrium concentrations between the protein–ligand complex and free protein are to be retained. Instrumental ESI source conditions that simultaneously maximize the relative ionization efficiency of the protein–ligand complex over free protein and minimize the protein–ligand complex dissociation during the ESI process and the transfer from atmospheric pressure to vacuum are generally specific for each protein–ligand system and should be established when an accurate equilibrium dissociation constant (K_D_) is to be determined via titration. In this paper, a straightforward and systematic approach for ESI source optimization is presented. The method uses statistical design of experiments (DOE) in conjunction with response surface methodology (RSM) and is demonstrated for the complexes between *Plasmodium vivax* guanylate kinase (*Pv*GK) and two ligands: 5′-guanosine monophosphate (GMP) and 5′-guanosine diphosphate (GDP). It was verified that even though the ligands are structurally similar, the most appropriate ESI conditions for K_D_ determination by titration are different for each.

**Electronic supplementary material:**

The online version of this article (doi:10.1007/s13361-016-1417-x) contains supplementary material, which is available to authorized users.

**Graphical Abstract:**
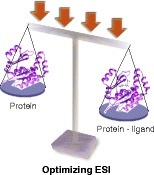
ᅟ

## Introduction

Binding interactions between ligands and proteins are crucial for a wide variety of biological processes and for the effectiveness of many therapeutic compounds. Among the methods available for the identification and characterization of noncovalent interactions between proteins and their ligands, electrospray ionization mass spectrometry (ESI-MS) offers advantages of high sensitivity, selectivity, simplicity, and speed [1–4]. With ESI-MS, native proteins and protein–ligand complexes can be transferred from solution to the gas phase as ions without changing their relative equilibrium concentrations [5, 6]. ESI-MS can probe specificity and stoichiometry of binding [7–10], protein conformational changes [11, 12], kinetics [13, 14], thermodynamics of binding [15, 16], and quantify equilibrium dissociation constants (K_D_) [17–25]. However, before establishing any correlation between the relative abundance of the gas-phase ions in the mass spectra and relative concentrations in solution, differences in ionization and detection efficiencies (response factors) between the free protein and protein–ligand complex, as well as processes such as nonspecific ligand binding and protein–ligand complex dissociation, need to be considered and, if possible, minimized.

To take into account any differences in ionization and detection efficiencies between the free protein and the protein–ligand complex, several methods that quantify their response factors (R) have been proposed [26–29]. The charge residue model (CRM) explains that high concentrations of ligand in the final droplet can lead to nonspecific interactions between free ligand and free protein, resulting in formation of nonspecific protein–ligand complexes on droplet evaporation. These complexes are indistinguishable from specific complexes originating from solution in the mass spectra [6, 30]. Based on the observation that nonspecific ligand binding is independent of the size and structure of the protein and follows a random distribution (Poisson distribution) [30–34], different strategies have been developed to correct the ESI mass spectra for the occurrence of nonspecific ligand binding [33–38].

Although most reports of K_D_ determination by ESI-MS recognize the importance of adjusting the instrumental source parameters to preserve the protein–ligand complexes formed in solution [39–43], none has provided a straightforward statistical approach for successful systematic ESI source optimization. Statistical design of experiments (DOE) and response surface methodology (RSM) have previously been shown to be useful to optimize signal sensitivity [44–49]. We have, for example, previously reported the use of a three-level fractional factorial design to maximize the absolute intensity of the noncovalent complex bCAII-ethoxzolamide and thus find the best conditions for screening natural product extracts against this protein [47].

Optimization of the ESI source was performed for the interactions between *Plasmodium vivax* guanylate kinase (*Pv*GK) and its natural substrates 5′-guanosine monophosphate (GMP) and 5′-guanosine diphosphate (GDP). The effects of determined preselected ESI source parameters on relative ion abundances, defined as the ratio of protein–ligand complex (1:1 stoichiometry) to free protein ion abundances (PL/P), were evaluated in inscribed central composite designs (CCIs). MS response analysis by RSM allowed the establishment of optimal ESI source conditions for K_D_ determination by titration, which was validated by competition experiments and literature K_D_ values.

## Experimental

### Materials

GMP and GDP were purchased from Sigma-Aldrich (Castle Hill, NSW, Australia). Ammonium acetate was purchased from Fluka (Castle Hill, NSW, Australia). GMP and GDP stock solutions were prepared in Milli-Q water (Millipore, North Ryde, NSW, Australia). For protein buffer exchange, Nalgene NAP-5 size G25, from GE Healthcare, (Parramatta, NSW, Australia) were used (purchased through Thermo Fisher Scientific Australia Pty Ltd, Scoresby, VIC, Australia).

### Protein Production

Full-length guanylate kinase with an N-terminal 6-histidine tag (23545 Da) was cloned, produced, and purified as previously described from *Plasmodium vivax* cDNA [50].

### ESI Source and FT-ICR Mass Spectrometry

All experiments were performed on a Bruker APEX III 4.7 T FT-ICR mass spectrometer equipped with an external Apollo ESI source (Supplementary Figure S1). The 14 experimental ESI source parameters that can be tuned are: sample flow rate, nebulizer gas (N_2_) pressure, end plate voltage, capillary voltage, drying gas (N_2_) flow rate, drying gas temperature, capillary exit voltage, skimmer 1 and skimmer 2 voltages, trapping and extracting voltages, and hexapole rf amplitude, hexapole DC offset voltage, and hexapole accumulation time (Supplementary Figure S1). All sample solutions were injected manually using a Cole-Parmer syringe pump. The nebulizer was off-axis and grounded. The glass capillary was 15 cm in length and had an internal diameter of 0.5 mm. Between the capillary exit and the skimmer 1, the background pressure was ~2 mbar. The ESI source housing, to where the ions are released after being accumulated inside the hexapole during a predetermined time, had a pressure of ~2 × 10^–6^ mbar. The analyzer was an Infinity cell.

*Pv*GK protein was buffer exchanged with 10 mM ammonium acetate buffer (pH 6.8) and stored in aliquots at –28 °C until needed. For optimization of the ESI source by DOE and RSM, working solutions of *Pv*GK:GMP (2:4.8 μM) and *Pv*GK:GDP (2:2.4 μM) were prepared prior to MS analysis by appropriately diluting the protein (thawed unassisted at room temperature) and the ligand in 10 mM ammonium acetate buffer (pH 6.8). For titration experiments, samples with a fixed concentration of P*v*GK (2 μM) and varying concentrations of ligand (0–28.8 μM of GMP and 0–7.2 μM of GDP) were prepared. For competition experiments, samples with a fixed concentration of P*v*GK and GMP (2 μM and 9.6 μM, respectively) and varying concentrations of GDP (0–9.6 μM) were prepared. Incubation time was 1 h at room temperature (23–25 °C).

The data were acquired in Bruker’s Xmass software. All spectra were recorded in positive ion mode, with a sum of 32 scans per acquisition and 512 k data points per transient. The obtained spectra were analyzed in mMass 5.5.0. software. The protein–ligand complex and free protein ion abundances were calculated as the sum of all charge states intensity peaks (*I*) normalized for charge state (*n*) (*∑ I(PL)*^*n+*^*/n* and *∑ I (P)*^*n+*^*/n*, respectively).

### Inscribed Central Composite Designs (CCI) for ESI Source Optimization

Optimization of the ESI source was carried out using central composite designs (CCD). Inscribed central composite designs (CCI) were chosen because the specified limits (higher and lower factor levels) corresponded to or were very close to truly instrumental limits for most of the selected factors. Each design included a full or fractional factorial portion created within the specified factor limits (by dividing the distance from the design center to the factor limits by α), from which the first order or linear effects could be estimated; a central point, from which curvature due to two-way interactions or quadratic terms could be evaluated; and a star portion with experimental points at the specified factor limits, from which the factors responsible for response surface curvature could be known. With this type of design, all factors were studied in five levels. The number of required experiments was given by *2*^*K-p*^*+ 2K + C*, where *K* is the number of factors included in the design, *p* the fraction of the full factorial design to be run, and *C* the number of replicates at the central point; *α* values were dependent on the number of variables and the desired design characteristics, such as rotatability and orthogonality. In this study, all CCIs were designed to be orthogonal and rotatable. All steps of the experimental design selection, statistical data analysis, and prediction of optimal factor settings were performed in R software [51], with the “rsm” package [52].

### K_D_ Determination by Titration

To determine *Pv*GK–GMP and *Pv*GK–GDP K_Ds_ by titration, the relative abundances of bound to total protein in the mass spectra were correlated to the relative equilibrium concentrations of bound to total protein in solution, as follows:

1 $$ \frac{{\displaystyle \sum }\ \mathrm{I}{\left(\mathrm{P}\mathrm{L}\right)}^{\mathrm{n}+}/\mathrm{n}\ }{{\displaystyle \sum }\ \mathrm{I}{\left(\mathrm{P}\right)}^{\mathrm{n}+}/\mathrm{n} + {\displaystyle \sum }\ \mathrm{I}{\left(\mathrm{P}\mathrm{L}\right)}^{\mathrm{n}+}/\mathrm{n}\kern0.5em } = \frac{\left[\mathrm{P}\mathrm{L}\right]}{{\left[\mathrm{P}\right]}_{\mathrm{t}}} $$

By plotting experimentally observed ratios between bound and total protein ion abundances against total concentration of ligand, K_D_ could be obtained as a parameter of a nonlinear least squares curve fitting [22]:

2 $$ \frac{{\displaystyle \sum }\ \mathrm{I}{\left(\mathrm{P}\mathrm{L}\right)}^{\mathrm{n}+}/\mathrm{n}}{{\displaystyle \sum }\ \mathrm{I}{\left(\mathrm{P}\right)}^{\mathrm{n}+}/\mathrm{n} + {\displaystyle \sum }\ \mathrm{I}{\left(\mathrm{P}\mathrm{L}\right)}^{\mathrm{n}+}/\mathrm{n}\kern0.5em } = \frac{\left(\left({\left[\mathrm{P}\right]}_{\mathrm{t}} + {\left[\mathrm{L}\right]}_{\mathrm{t}} + {\mathrm{K}}_{\mathrm{D}}\right)\ \hbox{-}\ \sqrt{{\left({\left[\mathrm{P}\right]}_{\mathrm{t}} + {\left[\mathrm{L}\right]}_{\mathrm{t}} + {\mathrm{K}}_{\mathrm{D}}\right)}^2\ \hbox{-}\ 4{\left[\mathrm{P}\right]}_{\mathrm{t}}{\left[\mathrm{L}\right]}_{\mathrm{t}}}\right)}{2{\left[\mathrm{P}\right]}_{\mathrm{t}}\ } $$

### K_D_ Determination by Competition

To determine *Pv*GK–GDP K_D_ by competition, the ion abundance of the reference protein –ligand complex with a known binding constant (*Pv*GK–GMP, with K_D_ determined by titration) was monitored in the presence and in the absence of the competitor ligand (GDP) and correlated to the corresponding solution equilibrium concentrations.

3 $$ \frac{{\left({\displaystyle \sum }\ \mathrm{I}{\left({\mathrm{PL}}_{\mathrm{ref}}\right)}^{\mathrm{n}+}/\mathrm{n}\ \right)}_{presence}}{{\left({\displaystyle \sum }\ \mathrm{I}{\left({\mathrm{PL}}_{\mathrm{ref}}\right)}^{\mathrm{n}+}/\mathrm{n}\ \right)}_{absence}} = \frac{{\left[{\mathrm{PL}}_{ref}\right]}_{presence}}{{\left[{\mathrm{PL}}_{ref}\right]}_{absence}} $$

By plotting the observed ratios between the reference protein –ligand complex in the presence and absence of competitor ligand as a function of total concentration of competitor ligand, the K_D_ of the competitor ligand could be obtained as a parameter of a nonlinear least squares curve fitting [23]:

4 $$ \frac{{\left({\displaystyle \sum }\ \mathrm{I}{\left({\mathrm{PL}}_{\mathrm{ref}}\right)}^{\mathrm{n}+}/\mathrm{n}\ \right)}_{presence}}{{\left({\displaystyle \sum }\ \mathrm{I}{\left({\mathrm{PL}}_{\mathrm{ref}}\right)}^{\mathrm{n}+}/\mathrm{n}\ \right)}_{absence}} = \frac{\frac{{\left[{L}_{ref}\right]}_t\left\{2\sqrt{\left({a}^2-3b\right)} \cos \left(\frac{\theta }{3}\right)-a\right\}}{3{K}_{D_{ref}}+\left\{2\sqrt{\left({a}^2-3b\right)} \cos \left(\frac{\theta }{3}\right)-a\right\}}}{\frac{\left({\left[P\right]}_t+{\left[{L}_{ref}\right]}_t+{K}_{D_{ref}}\right)-\sqrt{{\left({\left[P\right]}_t+{\left[{L}_{ref}\right]}_t+{K}_{D_{ref}}\right)}^2-4{\left[P\right]}_t{\left[{L}_{ref}\right]}_t}}{2}} $$

Where,

5 $$ a = {K}_{D_{ref}} + {K_D}_{{}_{comp}}+{\left[{L}_{ref}\right]}_t+{\left[{L}_{comp}\right]}_t-{\left[P\right]}_t $$

6 $$ b = {K_D}_{{}_{comp}}\left({\left[{L}_{ref}\right]}_t-{\left[P\right]}_t\right) + {K}_{D_{ref}}\left({\left[{L}_{comp}\right]}_t-{\left[P\right]}_t\right)+{K}_{D_{ref}}{K_D}_{{}_{comp}} $$

7 $$ c = - {K}_{D_{ref}}{K_D}_{{}_{comp}}{\left[P\right]}_t $$

8 $$ \theta = arc\  \cos \left(\frac{-2{a}^3+9 ab-27c}{2\sqrt{{\left({a}^2-3b\right)}^3}}\right) $$

## Results and Discussion

### Optimization of PvGK-GMP Complex Over Free PvGK Ion Abundances

Based on preliminary screening experiments (Supplementary Figure S2), nine out of the 14 ESI source parameters were selected for statistical optimization. Optimization of the ESI source was carried out in two stages because of the large number of variables. In the first, sample flow rate (120 to 630 μL/h), drying gas flow rate (20 to 60 L/min), drying gas temperature (100 to 150 °C) and nebulizer gas pressure (40 to 70 psi) were studied. In the second, capillary exit voltage (50 to 200 V), skimmer 1 voltage (15 to 25 V), skimmer 2 voltage (5 to 25 V), capillary voltage (–4000 to –6000 V), and end plate voltage (–2500 to –3500 V) were studied. It was assumed that if any interactions between factors included in the first and second CCI existed, these would not contribute significantly to the MS response. While the first stage CCI was carried out in 30 runs (2^4^ + 2 × 4 + 6), the second was carried out in 50 runs (2^5^ + 2 × 5 + 8).

After MS response collection, a second order model was used to fit the data using the least squares method. Different MS response transformations were evaluated, including square root, logarithmic, and squared transformations. Raw data was chosen last because residuals had a closer distribution to normality. Supplementary Tables S1 and S3 provide, for first and second stage CCI, respectively, the fitted model terms coefficients and respective statistical significances. Similarly, Supplementary Tables S2 and S4 show the ANOVA employed to evaluate the quality of the model fitted.

For the first stage CCI, a *P*-value of 5.82 × 10^–9^ was obtained, indicating the model was statistically significant. Moreover, the model had an adjusted R^2^ of 0.94 and a lack of fit *P*-value of 0.15 (indicating that lack of fit errors were due to random errors and were not considered statistically different from pure errors) (Supplementary Table S2). The residuals were normally distributed and equally dispersed for all predicted values, with no systematic trends observed. All variables had significant main effect on PL/P, with sample flow rate showing a significant quadratic term and no interaction with any other parameter (Supplementary Table S1).

Sample flow rate could be tuned independently of the settings of other variables. A threefold increase in sample flow rate from 120 μL/h up to around 400 μL/h favored *Pv*GK–GMP complex over free *Pv*GK ion abundances, after which the tendency was inverted.

**Figure 1:**
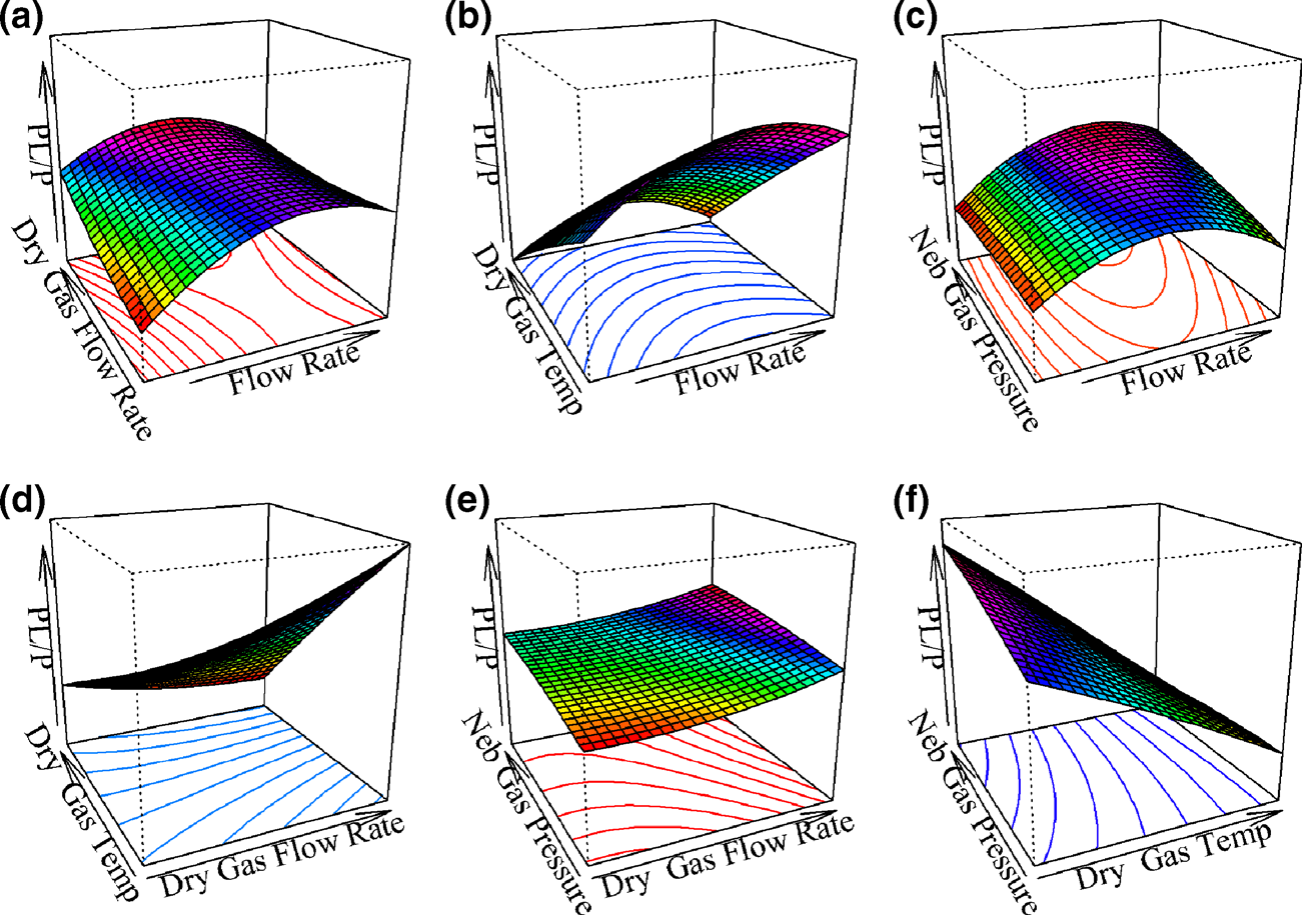
Three-dimensional response surface plots of the experimental region studied by first stage CCI, *Pv*GK-GMP system. Slices of the response surface are displayed for two variables at a time, with other variables fixed at the central point (flow rate 375 μL/h; drying gas flow rate 40 L/min; drying gas temperature 125 °C; nebulizer gas pressure 55 psi). **(a)** Effect of flow rate and drying gas flow rate. **(b)** Effect of flow rate and drying gas temperature. **(c)** Effect of flow rate and nebulizer gas pressure. **(d)** Effect of drying gas flow rate and drying gas temperature. **(e)** Effect of drying gas flow rate and nebulizer gas pressure. **(f)** Effect of drying gas temperature and nebulizer gas pressure

Drying gas temperature significantly interacted with drying gas flow rate. Thus, these parameters needed to be tuned together. The heated drying gas, N_2_, flows counter-current to the charged droplets trajectory and is very important for desolvation. Lower drying gas temperatures combined with higher drying gas flow rates provided the best PL/P. The three-dimensional response surface plots are presented in Figure 1.

**Figure 2:**
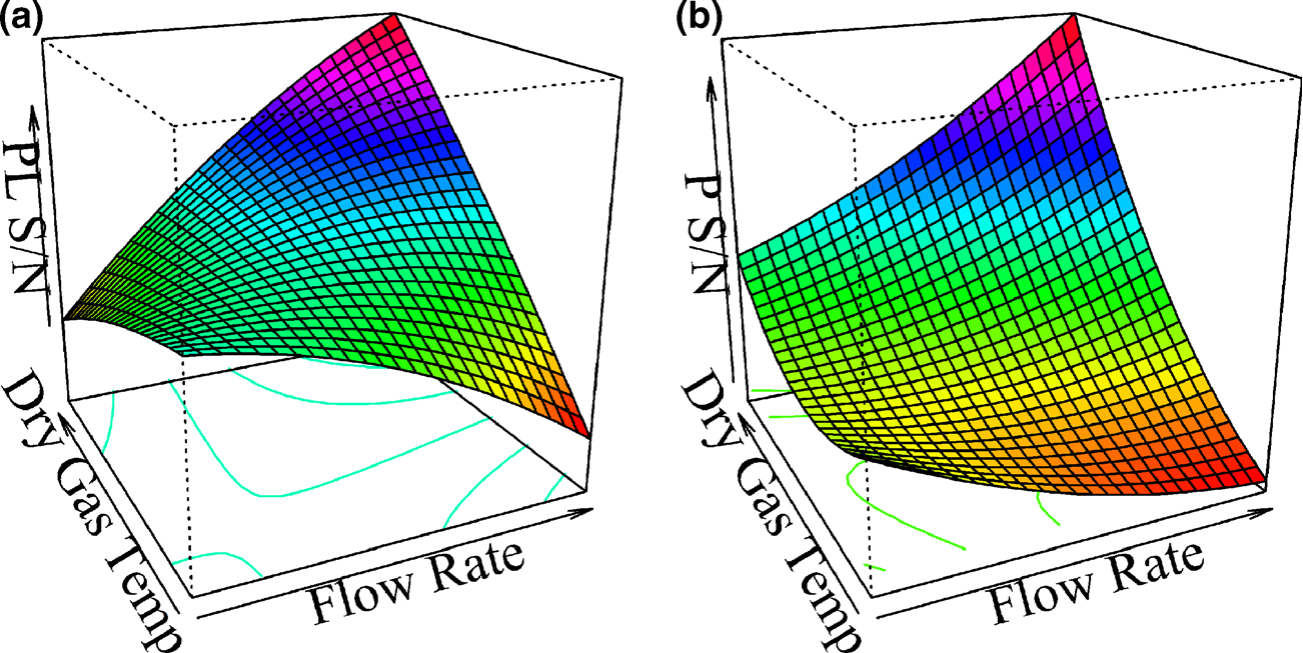
*Pv*GK-GMP complex (1:1 stoichiometry) **(a)** and free *Pv*GK **(b)** S/N as a function of flow rate (from 120 to 630 μL/h) and drying gas temperature (from 100 to 150 °C). The other variables were fixed at the central point (drying gas flow rate 40 L/min; nebulizer gas pressure 55 psi)

According to these results, better PL/P responses could theoretically be achieved outside the experimental region, especially if drying gas temperatures less than 100 °C were used with flow rates in the order of 400–420 μL/h. However, due to the observed trade-off between the relative ion abundances and intensities of both free protein and protein–ligand complex, such combination of factor levels could not be tested or used in practice. A second order model was also fitted to the signal-to-noise ratios (S/N) of free *Pv*GK and *Pv*GK–GMP complex with the only statistically significant interaction being between drying gas temperature and sample flow rate. The obtained three-dimensional surface plot for these two variables is shown in Figure 2 [(a) for *Pv*GK-GMP complex and (b) for free *Pv*GK]. It is interesting to note that at the lowest drying gas temperature (100 °C), the S/N of both free *Pv*GK and *Pv*GK–GMP complex decrease with increasing sample flow rate (probably due to a decrease in desolvation efficiency). However, if enough desolvation energy is provided by increasing the drying gas temperature, this trend is inverted (S/N increase with increasing sample flow rates).

Experimental verification confirmed that the maximum response was obtained at sample flow rate 400 μL/h, drying gas flow rate 45 L/min, drying gas temperature 105 °C, and nebulizer gas pressure 58 psi.

For the second stage CCI, the regression model was not significant (*P*-value of 0.73), even though the fit to the experimental data was good (lack of fit test was nonsignificant with a *P*-value of 0.66 and residuals had a normal distribution) (Supplementary Table S4).

Given the nonsignificant impact of capillary exit, skimmer 1, skimmer 2, end plate, and capillary voltages on relative ion abundances, these variables were used to simultaneously maximize the S/N of both free *Pv*GK and the *Pv*GK–GMP complex (1:1 stoichiometry). This approach involved the construction of a desirability function for each individual response and their combination by using the weighted geometric average to obtain an overall desirability function (R package “desirability” [53]) to establish capillary exit 126.5 V; skimmer 1 15.42 V; skimmer 2 15.55 V; end plate –3085 V; capillary voltage –4033 V. The improvement in S/N was 76% and 87% for free *Pv*GK and *Pv*GK-GMP complex, respectively.

The overall optimized conditions for *Pv*GK and the screening conditions are compared in Table 1. The screening conditions allowed the observation of protein and protein–ligand complexes under native conditions, even though relative solution-phase equilibrium concentrations between the protein–ligand complex and free protein might not be preserved. Indeed, the optimized conditions for *Pv*GK-GMP led to a relative ion abundance of 1.75, whereas screening conditions gave a relative ion abundance of 0.85.

**Table 1:** Optimized and Screening ESI Source Conditions

	Optimized conditions		Screening conditions
	*Pv*GK-GMP system	*Pv*GK-GDP system	
Sample flow rate (μL/h)	400	145	120
Drying gas flow rate (L/min)	45	38	40
Drying gas temperature (°C)	105	101	125
Nebulizer gas pressure (psi)	58	72	50
Capillary voltage (V)	–4033	–5100	–5000
End plate (V)	–3085	–3392	–3500
Capillary exit (V)	126.5	200	100
Skimmer 1 (V)	15.42	23.60	24.5
Skimmer 2 (V)	15.55	15.00	24
Hexapole RF amplitude (Hz)	600	600	600
Hexapole DC offset (V)	1.5	1.5	1.5
Hexapole accumulation time (s)	3	3	3
Trapping voltage (V)	23	23	23
Extracting voltage (V)	–10	–10	–10

### PvGK–GMP Titration Experiments and K_D_ Determination

**Figure 3:**
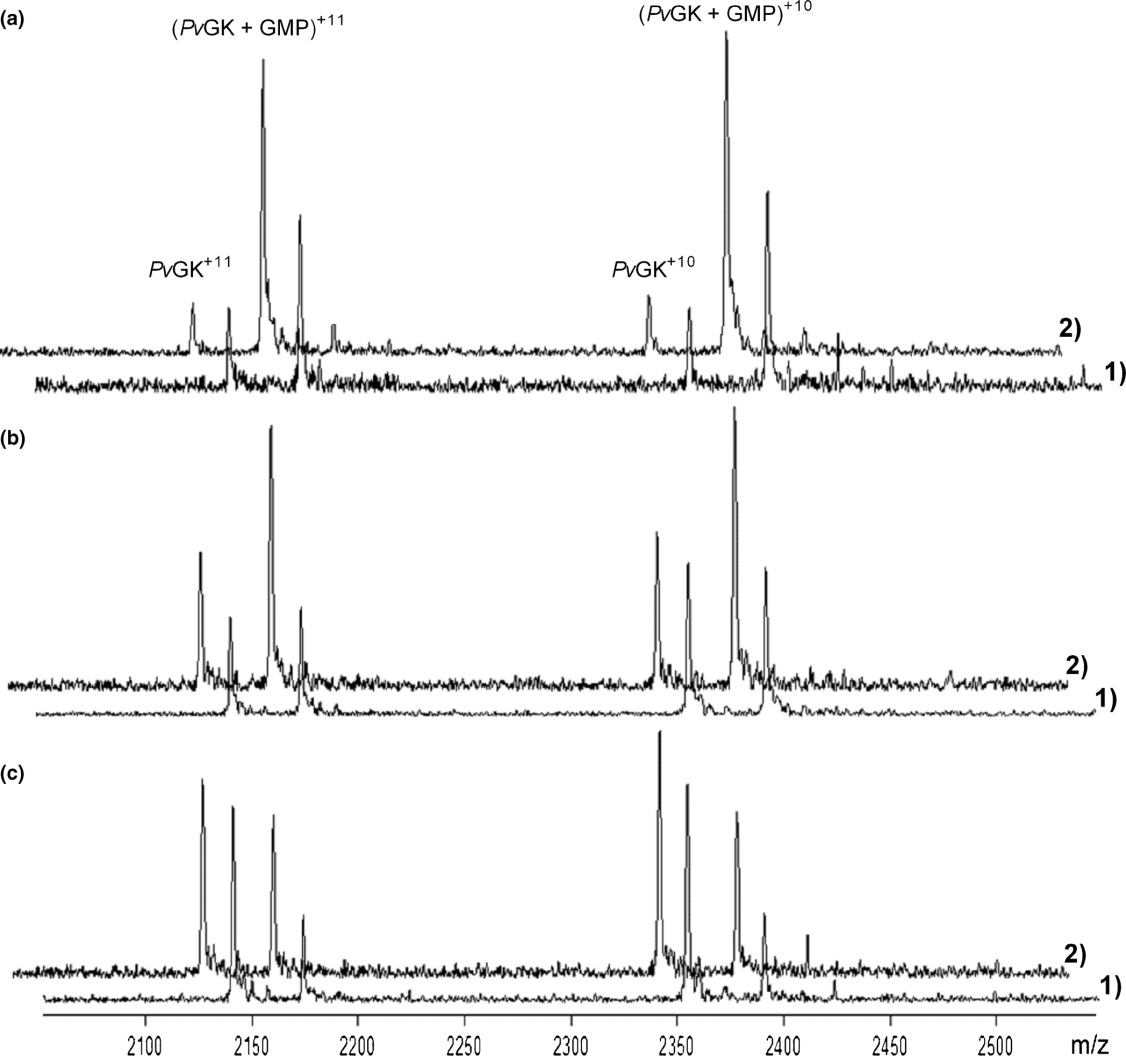
*Pv*GK-GMP titration experiments: representative spectra obtained from samples containing 2 μM *Pv*GK and three of the increasing concentrations of GMP [2.4 μM in **(c)**, 4.8 μM in **(b)** and 9.6 μM in **(a)**] acquired under ESI source optimized (2) and screening conditions (1)

Titration experiments demonstrated that under ESI optimized conditions the relative ion abundances were higher compared with screening conditions. Representative spectra obtained from samples containing *Pv*GK (2 μM) and three different concentrations of GMP (2.4, 4.8, and 9.6 μM) acquired under both optimized and screening ESI source conditions are shown and compared in Figure 3.

**Figure 4:**
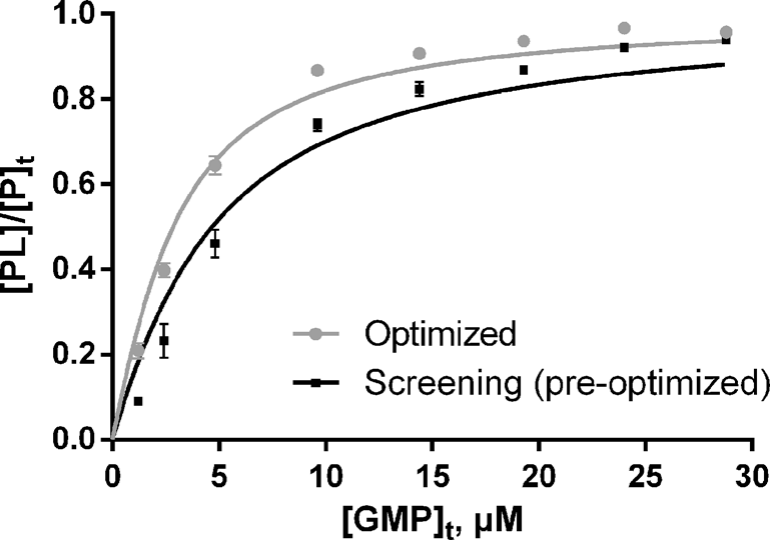
Fraction of bound protein ([PL]/[P]_t_) obtained under both ESI optimized and screening (pre-optimized) conditions plotted against total ligand concentration, GMP. The curves of best fit using the method of least squares with Equation (2) are present

At GMP concentrations of 9.6 μM and above, nonspecific protein–ligand binding was detected. The model developed by Daubenfeld et al. [37] was used to separate specific and nonspecific binding. The plot of the fraction of bound protein ([PL]/[P]_t_) versus total concentration of GMP and the best curve fit is shown in Figure 4. The K_D_ obtained after nonlinear least squares curve fitting using Equation (2) was 1.84 ± 0.11 μM and 3.67 ± 0.30 μM, respectively, for ESI source optimized and screening conditions.

The K_D_ obtained under ESI source optimized conditions for *Pv*GK–GMP was in good agreement with the results obtained for GMP binding to *Plasmodium falciparum* guanylate kinase (1.45 ± 0.05 μM at 25 °C, determined by isothermal titration calorimetry) [54]. By contrast, the K_D_ determined under screening conditions was an overestimate.

### Competition Experiments for PvGK-GDP K_D_ Determination

**Figure 5:**
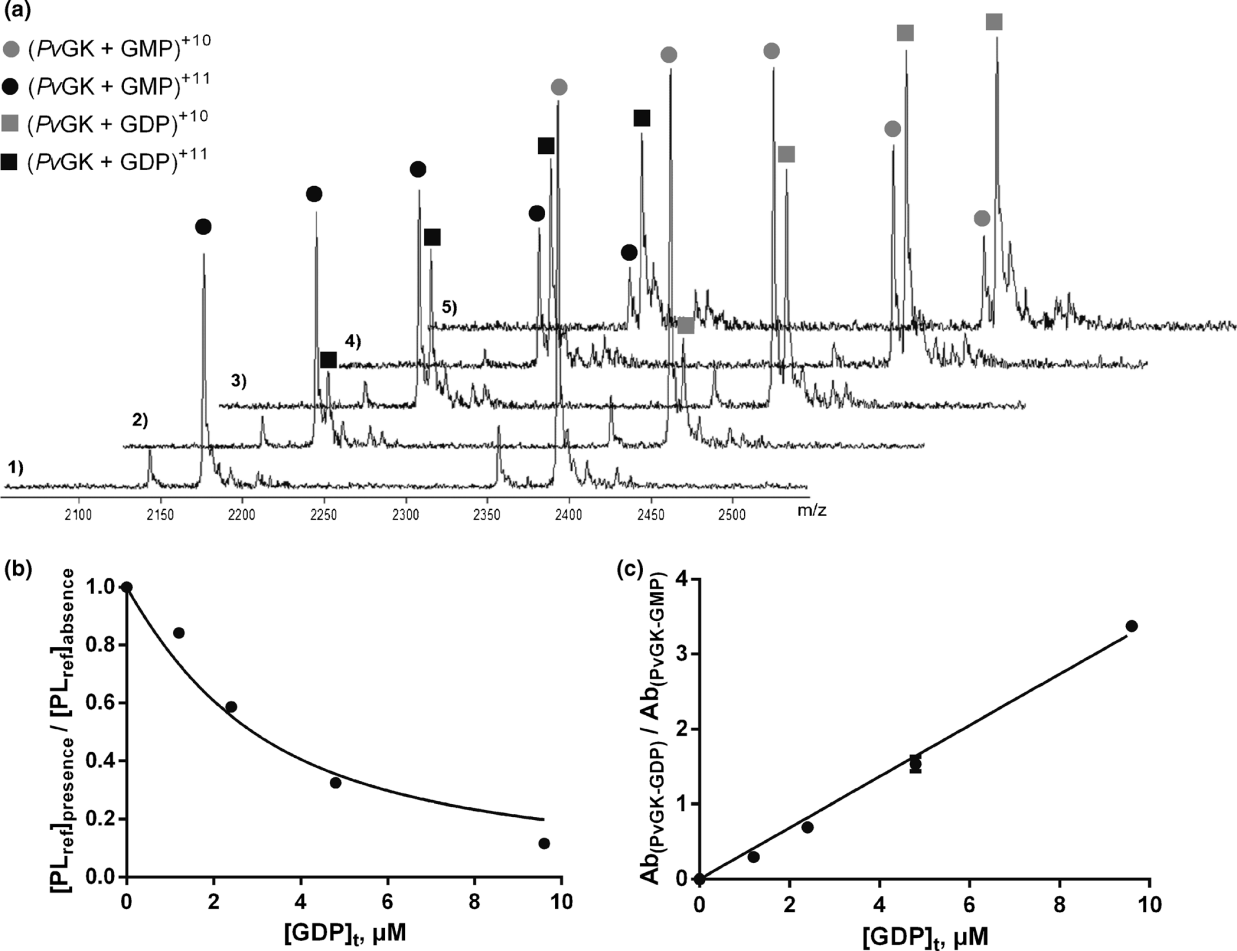
Competiton experiments: spectra obtained from samples containing 2 μM *Pv*GK, 9.6 μM GMP, and increasing concentrations of GDP [(0 μM in 1), (1.2 μM in 2), (2.4 μM in 3), (4.8 μM in 4), and (9.6 μM in 5)] are shown in **(a)**. Reference protein–ligand complex [*Pv*GK:GMP (2:9.6 μM)] concentration in the presence of competitor ligand (GDP) over reference protein–ligand complex concentration in the absence of competitor ligand is plotted as a function of total concentration of GDP in **(b)**. The solid line represents the best fit obtained using Equation (4). The linear relationship between the ion abundances of *Pv*GK-GDP complex over *Pv*GK-GMP complex and total GDP concentration is shown in **(c)**

Competition experiments revealed a clear competition between GMP and GDP for protein binding (Figure 5). As the concentration of GDP was increased in solution GMP was proportionally displaced from the protein, as can be observed from the decrease of *Pv*GK–GMP and increase of *Pv*GK-GDP complexes in Figure 5a and the linear relationship between the ratio of their ion abundances and total GDP concentration in Figure 5c. The degree of GMP displacement from the protein due to the presence of GDP was used to quantitatively estimate GDP K_D_ (Figure 5b)_._ The GDP K_D_ was estimated to be 0.31 ± 0.07μM. Given GDP has one more phosphate group compared with GMP, it contains more possibilities for electrostatic/Coulombic interactions with the protein and, thus, for binding more tightly. To cross-validate the ESI source optimization approach, this was independently optimized for the *Pv*GK–GDP system. The K_D_ determined by titration thereafter was compared with the K_D_ determined by competition.

### Optimization of PvGK-GDP Complex Over Free PvGK Ion Abundances

Optimization of the ESI source for the *Pv*GK–GDP system was carried out in two stages; in the first, sample flow rate (120 to 630 μL/h), drying gas flow rate (20 to 60 L/min), drying gas temperature (100 to 150 °C), and nebulizer gas pressure (30 to 80 psi) were studied. In the second, capillary exit voltage (50 to 200 V), skimmer 1 voltage (15 to 25 V), skimmer 2 voltage (5 to 25 V), capillary voltage (–4000 to –7000 V), and end plate voltage (–2500 to –3500 V) were studied.

The coefficients of the second-order model estimated with the raw MS responses of the first stage CCI is shown in Supplementary Table S5. Its ANOVA table (Supplementary Table S6) shows that the model was statistically significant (*P*-value 2.00 × 10^–9^), presenting an adjusted R^2^ of 0.85 and nonsignificant lack of fit (*P*-value 0.08). All variables except drying gas flow rate had significant main effect on PL/P. Similarly to the *Pv*GK–GMP system, sample flow rate had a significant quadratic term and a significant interaction with nebulizer gas pressure. Drying gas flow rate and drying gas temperature did not influence PL/P significantly. Confirmation experiments conducted at specified distances from the design center (0, 0.5, 1, 1.5) verified that maximum PL/P response could be obtained at sample flow rate 145 μL/h, drying gas flow rate 38 L/min, drying gas temperature 101 °C, and nebulizer gas pressure 72 psi.

**Figure 6:**
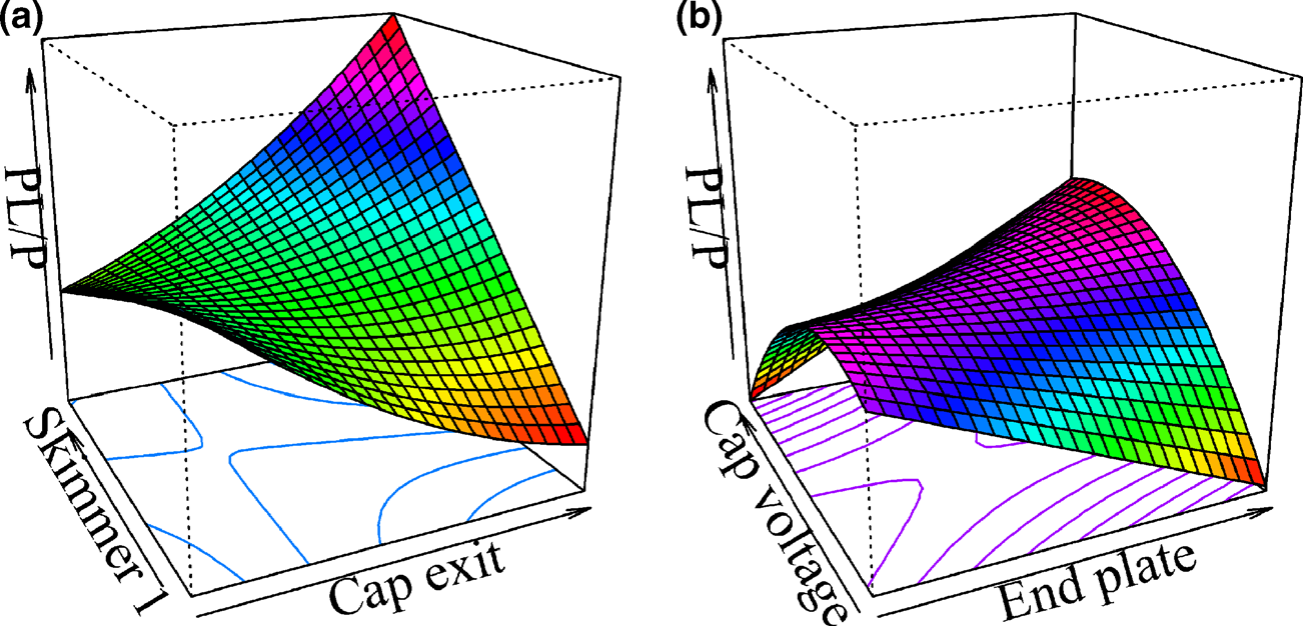
Three-dimensional response surface plots of skimmer 1 versus capillary exit **(a)** and capillary versus end plate voltages **(b)** (second stage CCI, *Pv*GK-GDP system). Other variables are fixed at the central point (skimmer 1, 20 V; skimmer 2, 15 V; end plate –3500 V; capillary voltage –5500 V; capillary exit 125 V)

For the second-stage CCI, the ANOVA demonstrates that the regression model estimated with the raw MS responses was statistically significant (*P*-value 2.27 × 10^–4^), presenting an adjusted R^2^ of 0.32 and nonsignificant lack of fit (*P*-value 0.17) (Supplementary Table S8). Contrary to what was observed for the *Pv*GK–GMP system, capillary exit had a significant main effect, as did its interaction with skimmer 1 (Supplementary Table S7). At lower skimmer 1 settings, an increase in capillary exit promoted a decrease in relative ion abundances, whereas at higher skimmer 1 values, an increase in capillary exit increased considerably relative ion abundances (Figure 6a). A similar effect was observed for a statistically significant interaction between end plate and capillary voltages (Figure 6b). For the *Pv*GK–GDP system no trade-off between relative ion abundances and S/N of both free *Pv*GK and *Pv*GK-GDP complex was found. We believe that the additional phosphate group of GDP contributes to the increased stability of the protein–ligand complex, thus tolerating higher voltages, which improve the declustering efficiency without promoting the complex dissociation. Experimental results confirmed that the maximum response could be obtained at capillary –5100 V, end plate –3392 V, capillary exit 200 V, skimmer 1, 23.60 V, and skimmer 2, 15.00 V. The overall optimized and screening conditions are compared in Table 1.

### PvGK–GDP Titration Experiments and K_D_ Determination

The K_D_ determined after nonlinear least squares curve fitting using Equation (2) was 0.30 ± 0.03 μM. The K_D_ was in excellent agreement with the K_D_ found by competition experiments (0.31 ± 0.07 μM). These results confirm the value of ESI source optimization by statistical DOE and RSM in order to improve the accuracy of K_D_ determinations by ESI-MS.

## Conclusions

A systematic approach, combining statistical DOE with RSM, simultaneously maximizes the relative ionization efficiency of a protein–ligand complex over free protein, minimizes the dissociation of protein–ligand complex and enables the establishment of appropriate ESI source settings for K_D_ determination by titration. If the fractional protein occupancy tends to one as ligand concentrations are increased, K_D_ can directly be determined by nonlinear least squares curve fitting using Equation (2). However, if nonspecific ligand binding is observed, this needs to be considered before K_D_ determination. In this study, the model introduced by Daubenfeld et al. [37], which explains ligand binding as a convolution of two different statistical distributions (a binomial distribution for specific ligand binding and a Poisson distribution for nonspecific ligand binding), was used to compute the contributions from only specific ligand binding. If the fractional protein occupancy does not tend to one even after ESI source optimization, we propose that the horizontal asymptote on the right side is determined and used to correct the experimental data, in a similar way presented by Jaquillard et al. [25]. Here, the assumption that protein–ligand complex dissociation is only dependent on its gas-phase stability under vacuum and thus the type of intermolecular interactions involved in binding is made. Hence, a constant correction factor can be applied to the experimental fraction of bound protein obtained over the range of ligand concentrations used for the titration experiment.

**Scheme 1:**
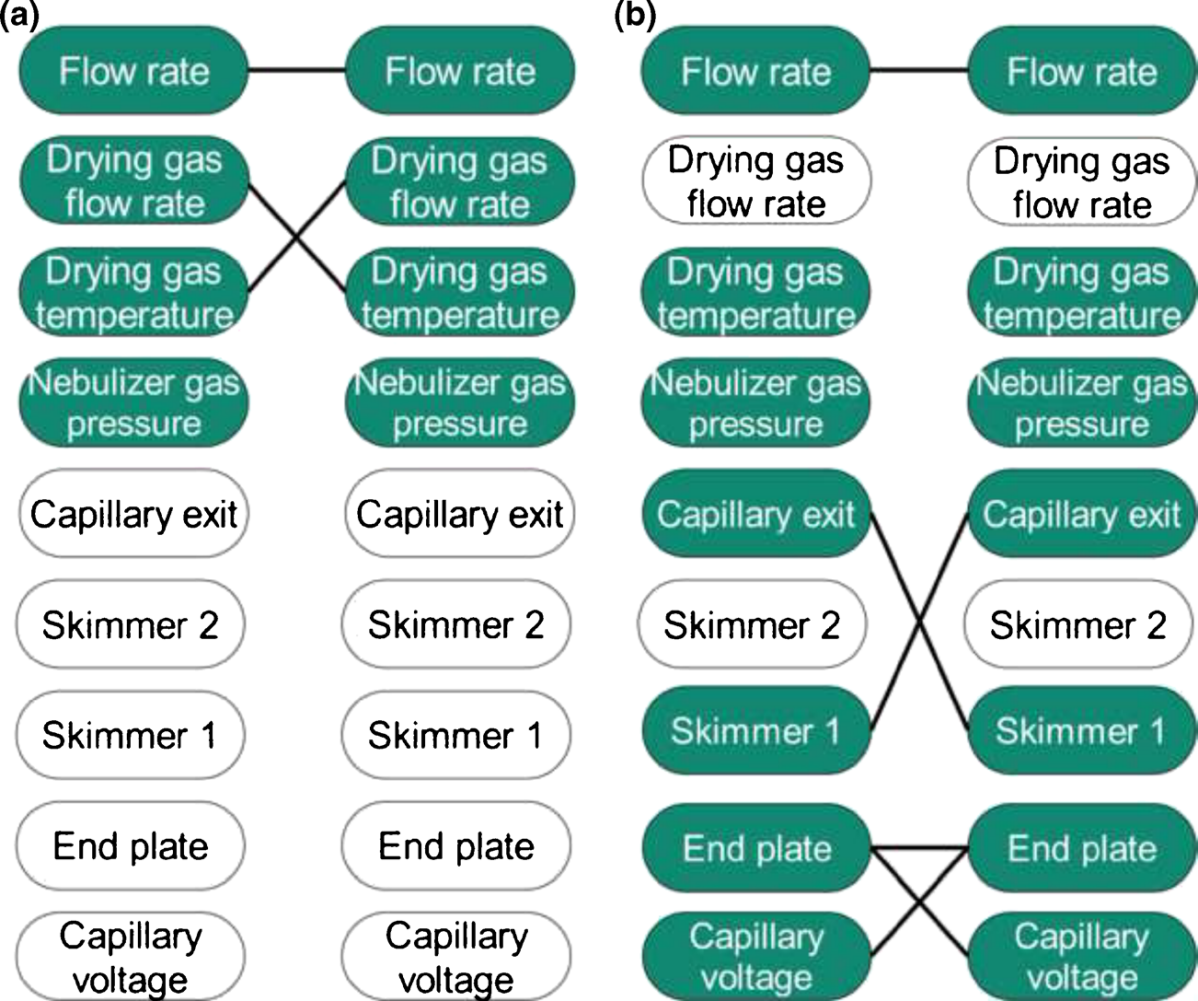
Summary of ESI source parameters studied by statistical DOE and RSM and their influence on relative ion abundances for **(a)** *Pv*GK-GMP system and **(b)** *Pv*GK-GDP system: parameters inside white boxes had no significant impact on relative ion abundances; parameters inside colored boxes had significant main effect on relative ion abundances; connecting lines denote significant interactions or quadratic effects

The optimization was demonstrated for the noncovalent interactions between *Pv*GK and its natural substrates GMP and GDP. The results show that the ESI conditions, which better retain relative solution-phase equilibrium concentrations between a protein–ligand complex and free protein, depend on the protein–ligand system itself (Scheme 1). These results also showed that if ESI source instrumental conditions are carefully chosen, the accuracy of K_D_ determinations by ESI-MS can be improved.

## Electronic supplementary material

Below is the link to the electronic supplementary material.

ESM 1 (PDF 588 kb)
