# Supplementary material for: Optimization of Electrospray Ionization by Statistical Design of Experiments and Response Surface Methodology: Protein–Ligand Equilibrium Dissociation Constant Determinations
Source: J Am Soc Mass Spectrom. 2016 May 25;27:1520–30. doi: 10.1007/s13361-016-1417-x (PMC4972871; doi:10.1007/s13361-016-1417-x)
Supplement: Supplementary file 1 — (PDF 588 kb) [file 13361_2016_1417_MOESM1_ESM.pdf]

Journal of the American Society for Mass Spectrometry

Supplementary Information for:

Optimization of Electrospray Ionization by Statistical Design of Experiments and Response Surface Methodology: Protein - Ligand Equilibrium Dissociation Constant Determinations

Liliana Pedro[1], Wesley C Van Voorhis[2], Ronald J Quinn[1]

[1]Eskitis Institute for Drug Discovery, Griffith University, Brisbane, Queensland, Australia.
[2]Department of Medicine, University of Washington, Seattle, WA, USA.

Correspondence to: Ronald J Quinn; e-mail address: r.quinn@griffith.edu.au

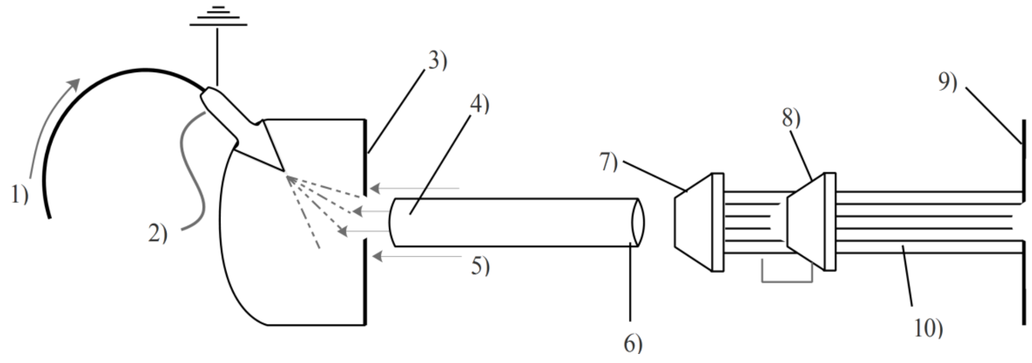

Fig. S1 External Apollo ESI source. A sample solution is sprayed at a determined flow rate (1)) into the source chamber by the nebulizer. With the help of a nebulizer gas (2)) and a potential difference between the inner chamber wall and the end plate (3)), charged droplets are generated. As these droplets drift through the air toward the opposing electrodes (end plate and capillary (4))), they are desolvated by a curtain of heated gas flowing counter current at a determined flow rate and temperature (5)). As solvent evaporates, droplets undergo fission and, ultimately, gas-phase ions are produced. Ions are conducted throughout the glass capillary towards the vacuum region. From the capillary exit (6)), ions pass through the skimmer 1 (7)), pre-hexapole and skimmer 2 (8)) and enter into the main RF ion guide, the hexapole (10)). Inside the hexapole, ions are trapped and accumulated by applying a voltage to the trap/extract electrode (9)). After a pre-determined period, the voltage of the trap/extract electrode drops and remains low for a pre-determined time. During this time, ions emerge from the hexapole. After, the next cycle of ion accumulation begins

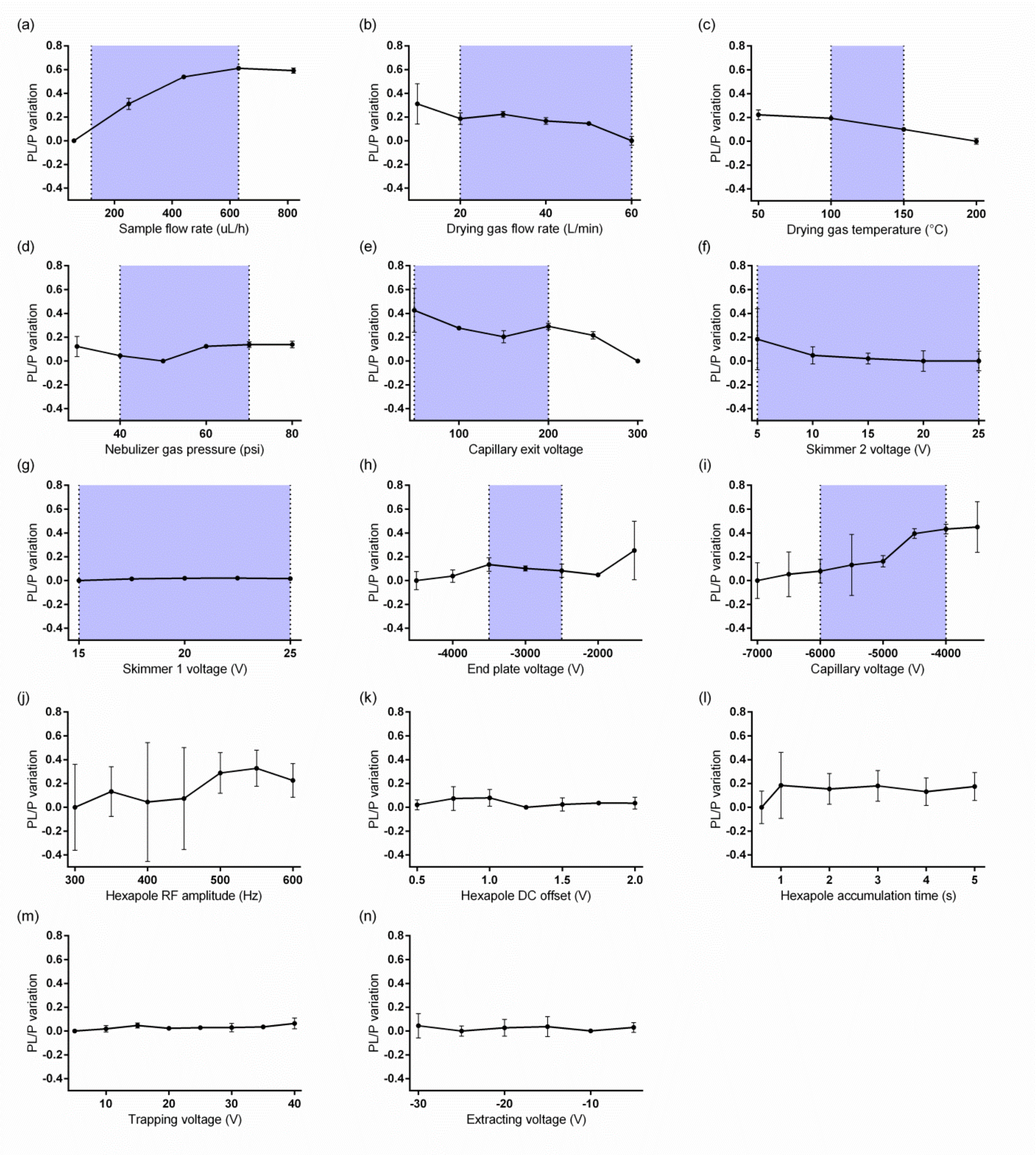

Fig. S2 Screening of ESI source parameters for the *Pv*GK-GMP system. The variation of relative ion abundances (PL/P), calculated as the difference between the actual and minimum observed value, is plotted as a function of sample flow rate (a), drying gas flow rate (b), drying gas temperature (c), nebulizer gas pressure (d), capillary exit (e), skimmer 2 (f), skimmer 1 (g), end plate (h), capillary voltage (i), hexapole RF amplitude (j), hexapole DC offset (k), hexapole accumulation time (l),

trapping voltage (m) and extracting voltage (n). Nine parameters were selected for further analysis by statistical DOE. These included the factors with relevant PL/P variation and the factors likely to interact significantly with them. The lower and higher factor levels were chosen taking into account relative ion abundances, signal stabilities and signal intensities. These are represented as vertical dashed lines, being the filled region between them the studied experimental region

Table S1 Estimated coefficients for the second-order response surface model fitted to the first stage Inscribed Central Composite Design (*Pv*GK-GMP system ESI source optimization)

	**Estimate**	**Std. Error**	**t-statistic**	**p-value**	**Signif. Code**[a]
Intercept	2.0471	0.0377	54.3518	< 2.2e-16	***
Flow rate	0.1526	0.0377	4.0521	0.0010	**
Drying gas flow rate	0.1338	0.0377	3.5525	0.0029	**
Drying gas temperature	-0.7560	0.0377	-20.0726	0.0000	***
Nebulizer gas pressure	0.1086	0.0377	2.8843	0.0113	*
Flow rate: Drying gas flow rate	-0.1537	0.0923	-1.6664	0.1164	
Flow rate: Drying gas temperature	-0.1223	0.0923	-1.3256	0.2048	
Flow rate: Nebulizer gas pressure	0.1298	0.0923	1.4073	0.1797	
Drying gas flow rate: Drying gas temperature	-0.2930	0.0923	-3.1759	0.0063	**
Drying gas flow rate: Nebulizer gas pressure	-0.0067	0.0923	-0.0722	0.9434	
Drying gas temperature: Nebulizer gas pressure	-0.1832	0.0923	-1.9858	0.0656	.
Flow rate ^2	-0.4257	0.0705	-6.0424	0.0000	***
Drying gas flow rate ^2	0.0795	0.0705	1.1287	0.2768	
Drying gas temperature ^2	-0.0411	0.0705	-0.5838	0.5680	
Nebulizer gas pressure ^2	-0.0348	0.0705	-0.4946	0.6281	

[a] Significance Codes: 0 < ‘***’ < 0.001 < ‘**’ < 0.01 < ‘*’ < 0.05 < ‘.’ < 0.1 < ‘ ’ < 1.

Table S2 ANOVA table for the second-order response surface model fitted to the first stage Inscribed Central Composite Design (*Pv*GK-GMP system ESI source optimization)

Source of Variance	Sum of Squares	Df	Mean Square	F value	p-value
**Model**	4.2671	14	0.3048	35.8140	5.82E-09
First Order	3.7469	4	0.93672	110.0674	6.31E-11
Two-way interactions	0.1749	6	0.02915	3.4249	0.0247
Pure Quadratic	0.3453	4	0.08633	10.1441	0.0004
**Residuals**	0.1277	15	0.00851		
Lack of fit	0.1073	10	0.01073	2.6389	0.1479
Pure error	0.0203	5	0.00407		
**Total**	4.3948	29			
**R^2**	0.9710				
**Adjusted R^2**	0.9438				

Table S3 Estimated coefficients for the second-order response surface model fitted to the second stage Inscribed Central Composite Design (*Pv*GK-GMP system ESI source optimization)

	**Estimate**	**Std. Error**	**t-statistic**	**p-value**	**Signif. Code[a]**
Intercept	0.5935	0.0351	16.8865	<2e-16	***
Capillary exit	-0.0280	0.0361	-0.7761	0.4440	
Skimmer 2	0.0181	0.0361	0.5008	0.6203	
Skimmer 1	0.0337	0.0361	0.9329	0.3586	
End Plate	-0.0074	0.0361	-0.2053	0.8387	
Capillary Voltage	0.0443	0.0361	1.2267	0.2298	
Capillary exit: Skimmer 2	-0.0335	0.0994	-0.3373	0.7384	
Capillary exit: Skimmer 1	0.0140	0.0994	0.1411	0.8888	
Capillary exit: End Plate	0.0840	0.0994	0.8448	0.4052	
Capillary exit: Capillary Voltage	-0.1486	0.0994	-1.4954	0.1456	
Skimmer 2: Skimmer 1	-0.1535	0.0994	-1.5449	0.1332	
Skimmer 2: End Plate	0.0111	0.0994	0.1119	0.9117	
Skimmer 2: Capillary Voltage	-0.0840	0.0994	-0.8447	0.4052	
Skimmer 1: End Plate	-0.1322	0.0994	-1.3306	0.1937	
Skimmer 1: Capillary Voltage	0.1456	0.0994	1.4647	0.1538	
End Plate: Capillary Voltage	-0.0194	0.0994	-0.1949	0.8468	
Capillary exit ^2	0.0213	0.0759	0.2812	0.7806	
Skimmer 2^2	0.0318	0.0759	0.419	0.6783	
Skimmer 1^2	-0.0421	0.0759	-0.5556	0.5827	
End Plate ^2	-0.0652	0.0759	-0.8592	0.3973	
Capillary Voltage ^2	-0.0594	0.0759	-0.7837	0.4396	

[a] Significance Codes: 0 < '***' < 0.001 < '**' < 0.01 < '*' < 0.05 < '.' < 0.1 < ' ' < 1

Table S4 ANOVA table for the second-order response surface model fitted to the second stage Inscribed Central Composite Design (*Pv*GK-GMP system ESI source optimization)

Source of Variance	Sum of Squares	Df	Mean Square	F value	p-value
**Model**	0.1543	20	0.0077	0.7701	0.7251
First Order	0.0328	5	0.0066	0.6541	0.6608
Two-way interactions	0.1017	10	0.0102	1.0151	0.4549
Pure Quadratic	0.0198	5	0.0040	0.3961	0.8474
**Residuals**	0.2905	29	0.0100		
Lack of fit	0.2096	22	0.0095	0.8248	0.6626
Pure error	0.0809	7	0.0116		
**Total**	0.4448	49			
**R^2**	0.3469				
**Adjusted R^2**	-0.1036				

Table S5 Estimated coefficients for the second-order response surface model fitted to the first stage Inscribed Central Composite Design (*Pv*GK-GDP system ESI source optimization)

	**Estimate**	**Std. Error**	**t-statistic**	**p-value**	**Signif. Code[a]**
Intercept	1.8697	0.0428	43.7141	<2.20E-16	***
Flow rate	-0.1824	0.0605	-3.0154	0.0062	**
Drying gas flow rate	-0.0760	0.0605	-1.2571	0.2213	
Drying gas temperature	-0.6959	0.0605	-11.5046	0.0000	***
Nebulizer gas pressure	0.2401	0.0605	3.9692	0.0006	***
Flow rate ^2	0.2569	0.1111	2.3122	0.0301	*
Nebulizer gas pressure ^2	0.1838	0.1111	1.6543	0.1116	

[a] Significance Codes: 0 < '***' < 0.001 < '**' < 0.01 < '*' < 0.05 < '.' < 0.1 < ' ' < 1.

Table S6 ANOVA table for the second-order response surface model fitted to the first stage Inscribed Central Composite Design (*Pv*GK-GDP system ESI source optimization)

Source of Variance	Sum of Squares	Df	Mean Square	F value	p-value
**Model**	3.6465	6	0.6078	27.68	2.00E-09
First Order	3.4857	4	0.8714	39.6956	5.23E-10
Pure Quadratic	0.1608	2	0.08038	3.6616	0.04163
**Residuals**	0.5049	23	0.02195		
Lack of fit	0.469	18	0.02606	3.6295	0.07945
Pure error	0.0359	5	0.00718		
**Total**	0.1540	29			
**R^2**	0.8784				
**Adjusted R^2**	0.8466				

Table S7 Estimated coefficients for the second-order response surface model fitted to the second stage Inscribed Central Composite Design (*Pv*GK-GDP system ESI source optimization)

	**Estimate**	**Std. Error**	**t-statistic**	**p-value**	**Signif. Code[a]**
Intercept	1.6029	0.4728	33.9049	<2.2E-16	***
Capillary exit	0.2570	0.0773	3.3245	0.0019	**
Skimmer 2	0.1069	0.0773	1.3832	0.1743	
Skimmer 1	0.7317	0.0773	0.9464	0.3496	
End Plate	0.0341	0.0773	0.4414	0.6612	
Capillary Voltage	0.0468	0.0773	0.6056	0.5482	
Capillary exit: Skimmer 1	0.5572	0.2130	2.6161	0.0123	*
End Plate: Capillary Voltage	0.4341	0.2130	2.0383	0.0482	*
Skimmer 1^2	0.1524	0.1593	0.9565	0.3445	
End Plate ^2	-0.3796	0.1593	-2.3832	0.0220	*

[a] Significance Codes: 0 < '***' < 0.001 < '**' < 0.01 < '*' < 0.05 < '.' < 0.1 < ' ' < 1

Table S8 ANOVA table for the second-order response surface model fitted to the second stage Inscribed Central Composite Design (*Pv*GK-GDP system ESI source optimization)

Source of Variance	Sum of Squares	Df	Mean Square	F value	p-value
**Model**	1.4957	9	0.1662	3.6110	2.27E-04
First Order	0.6637	5	0.1327	2.8846	0.0257
Two-way interactions	0.3149	1	0.3149	6.8440	0.0125
Two-way interactions	0.1912	1	0.1912	4.1545	0.0482
Pure Quadratic	0.3259	2	0.1629	3.5407	0.0384
**Residuals**	1.8406	40			
Lack of fit	1.6627	33	0.0504	1.9821	0.1757
Pure error	0.1779	7			
**Total**	3.3363	80			
**R^2**	0.4483				
**Adjusted R^2**	0.3242				
